# Statistical issues and challenges in immuno-oncology

**DOI:** 10.1186/2051-1426-1-18

**Published:** 2013-10-21

**Authors:** Tai-Tsang Chen

**Affiliations:** 1 Department of Global Biometric Sciences, Bristol-Myers Squibb, Wallingford, CT, USA; 2 Department of Biostatistics, Columbia University, New York, NY, USA

**Keywords:** Immunotherapy, Study design, Long term survivors, Delayed clinical effect, Group sequential method, Immune-related response criteria, Immune-related adverse events, Immune-mediated adverse reactions

## Abstract

**Background:**

The development of immuno-oncologic agents poses unique challenges, namely that both efficacy and safety profiles differ from previously characterized cytotoxic and pathway-specific agents. In addition, exponential distribution is usually assumed in study designs with time-to-event endpoints such as overall survival or progression-free survival. This assumption might lead to wrong estimates of study duration and statistical power if the phenomena of long term survival and delayed clinical effects are present. The aim here was to evaluate the magnitude of the impact caused by the violation of this assumption, and to describe new ways of analyzing efficacy and safety of immuno-oncologic agents.

**Methods:**

Monte Carlo simulation was implemented to explore the impact of long term survivors and delayed treatment effect on study power and trial duration. Scenarios with various combinations of long term and delayed treatment effects were considered. Study power and duration were evaluated based on 10000 randomly generated trial data sets. The utility of group sequential study designs was discussed. A new set of immune-related response criteria (irRC) was considered for efficacy analysis. Two new methods for identifying adverse events, termed immune-related adverse events (irAE) and immune-mediated adverse reactions (imAR) were described. The key features of the safety profiles derived using these two methods were similar. Both methods were aimed at identifying inflammatory adverse events caused by immunotherapies.

**Results:**

The presence of long term survivors usually lengthened the study duration. Depending on the treatment effect post survival curve separation, delayed clinical effect in general led to a loss of power. The irRC offered a new way of identifying clinical responses. Both safety analyses demonstrated higher sensitivity of identifying adverse events of immune system origin.

**Conclusion:**

This simulation study showed the importance of accounting for the delayed treatment effect and long term survivors when these phenomena were expected. Interim analyses for the purpose of stopping the study for either positive or futile outcome should be implemented with caution in immuno-oncology trials. The new efficacy analysis offered a potential new way of assessing signs of activity in immunotherapies. While the irAE method facilitated prompt and effective management of adverse events, the imAR method captured truly immune-related events.

## Background

Innovative research in recent years has led to the discovery of many promising targeted anti-cancer agents, including selective or multi-targeted inhibitors of tyrosine kinases, signal transduction, angiogenesis, or matrix metalloproteinase as well as targeted immunotherapies such as monoclonal antibodies, T cell infusion, and cancer vaccines. The varying mechanisms of action introduced by these novel agents challenge the researchers to reconsider whether the conventional efficacy and safety analyses as well as trial designs adequately address these new mechanisms under study.

Cytotoxic and cytostatic agents are classified based on their mechanism. Classical cytotoxic agents derive their anti-tumor activity from dose-dependent rapid cell kill. This mechanism of action, nevertheless, usually results in undesired toxicities due to the lack of selectivity between normal and cancerous cells. In contract, cytostatic compounds are agents that suppress cellular growth and division. These compounds are usually characterized by minimal or less severe toxicity, prolonged duration of the treatment, anti-tumor activities at dose levels potentially lower than the maximum tolerated dose (MTD), and inhibition of tumor growth with absence of or minimum tumor shrinkage.

Immunotherapies, on the other hand, stimulate the patient’s own immune system to fight against cancers by targeting antigens expressed on cancer cells. More than any other discovery, monoclonal antibodies (mAbs) have enabled us to identify and manipulate molecules regulating the immune system [1]. They represent a significant subset of immunotherapy agents being used to treat cancers. One such example is ipilimumab, a fully human monoclonal antibody (IgG1) that blocks cytotoxic T lymphocyte-associated protein 4 (CTLA-4, also known as CD152) to promote immunity. Either alone or in combination with dacarbazine (DTIC), ipilimumab has demonstrated a statistically significant improvement in overall survival (OS) in two phase III randomized controlled trials in patients with previously treated and treatment naïve metastatic melanoma [2,3].

The development process of cytotoxic and cytostatic agents has been well established. The design of clinical trials progresses from the early phase studies with the goal of assessing safety to later phase studies evaluating efficacy [4]. Phase I studies aim to assess the side-effects, the pharmacokinetics of an agent and to determine the MTD as well as the recommended phase II dose for subsequent phase II studies to evaluate the potential anti-tumor effect. If the agents under investigation are deemed sufficiently efficacious to warrant further investigation, larger phase III randomized clinical trials are conducted to confirm the clinical benefit. This traditional development paradigm can also be used to assess novel cytostatic agents and immunotherapies, especially those that may reduce the total tumor burden. However, with potentially different toxicity profiles and different mechanisms that induce anti-tumor activity, conventional endpoints and/or study designs may not allow optimal means for evaluating clinical efficacy and safety for these new classes of cancer therapies.

In this article, the focus will be on the late stage oncology drug development and the issues introduced by immuno-oncologic agents. First, several immunotherapies have been demonstrated to show delayed clinical effect [2,5-7], in contrast to cytotoxic chemotherapy from which patients usually derive early benefit. This phenomenon reduces the statistical power to differentiate between two treatment arms [8].

In addition, therapies for certain cancer types are believed to induce a subset of long term survivors, such as melanoma [2,3,9-12], head and neck cancer [13] and myeloid leukemia. Since the introduction of imatinib mesylate and dasatinib, the long term survival (> 5 years) rate of chronic myeloid leukemia patients has been reported as high as 70% [14,15]. Long term follow-up demonstrated that treatment with ipilimumab in patients with treatment-naïve and previously treated metastatic melanoma yielded survival rates of 20% beyond four and five years [16,17]. A long term survival rate in the range of 30% to 50% was also observed in Interferon and pegylated interferon *α*-2b in the treatment of adjuvant melanoma. In this setting, this long-term phenomenon can also be observed in other time to event endpoints such as recurrence-free survival (RFS) and distant metastases free survival (DMFS). Since a subset of the patients in the study is no longer at risk of progression or death, this implies that the study duration could be substantially prolonged. In some occasions, delayed clinical effect and long term survival were present in the same study. For instance, a randomized double blind phase III study comparing ipilimumab with and without gp100 vaccine vs. gp100 vaccine alone in patients with pre-treated advanced melanoma has shown that the Kaplan-Meier survival curves did not separate until approximately 4 months with a survival probability leveling off at 20% [2] in the experimental arms.

Another important aspect of randomized studies is the use of interim analyses. The implementation of the group sequential designs has become standard practice in phase III pivotal studies to allow early study termination in the face of either positive or negative outcome. Data have suggested that this standard approach warrants further reconsideration with immuno-oncologic products. For example, a phase III study comparing tremelimumab to standard-of-care in advanced melanoma was terminated early due to futility as the interim analysis failed to demonstrate an overall survival benefit during the follow-up [18].

The irRC guidelines for evaluating immune response were developed based on the conventional WHO or RECIST criteria to capture the unique characteristics exhibited by immuno-oncology agents. Finally, the conventional safety analysis in the ipilimumab studies revealed that the adverse event rates were similar between treatment arms. It was clear that to accurately characterize the safety profile of immuno-oncology agents such as ipilimumab, it was necessary to develop new methods to increase the sensitivity and specificity of capturing the events that were truly of immune system origin.

Here the main goal was to raise the awareness in both the statistical and clinical communities the importance of fully understanding the characteristics of the compounds under investigation, especially immuno-oncologic agents, in order to determine the optimal trial design and selection of the best endpoints, as well as the most appropriate efficacy and safety analysis approaches.

## Methods

### Long term survival and delayed clinical effect

**Figure 1:**
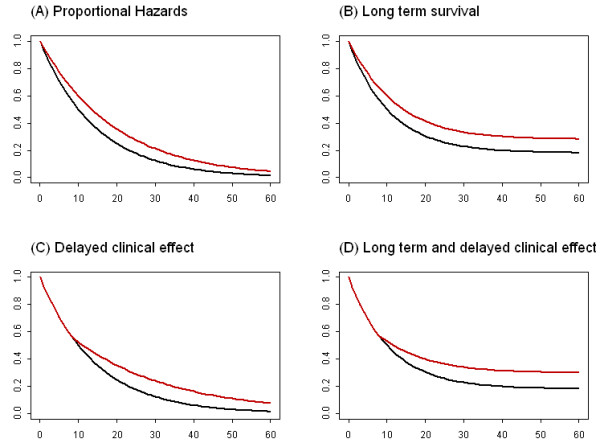
**Graphical presentation of Kaplan-Meier survival curves with various combinations of long term survival and delayed clinical effect.** The following plots show four hypothetical scenarios of overall survival outcome where the red and black curves represent novel immuno-oncologic agent and a control treatment respectively. **(A)** Conventional proportional hazards model with exponential assumption; **(B)** Proportional hazards model with long term survival; **(C)** Non-proportional hazards model with delay clinical effect; **(D)** Non-proportional hazards cure rate model with long term and delayed effects.

In a study with time-to-event endpoints, we are interested in the time interval between the entry of the study and an event, e.g., the interval between time of randomization and death or disease progression in a comparative study. The study is usually designed based on exponential distribution assumption in which we assume that anything which affects the hazards does so by the same ratio at all times, i.e., proportional hazards. This implies the clinical effect of the experimental arm over the control is observed from the beginning and the survival curves will eventually drop down to zero survival probability (Figure 1A). In general, this assumption is not unreasonable, nevertheless, the following two deviations will potentially lead to an underestimation of study duration or loss of statistical power.

Due to recent advancement in oncology therapy, a proportion of patients are expected to be cured (or non-susceptible) in certain diseases, that is to remain alive or disease-free even after long follow-ups. This phenomenon is usually observed in Kaplan-Meier curves with non-zero tail probabilities (Figure 1B, D). Since no one can be really "cured" of death, the term "cure" refers to one who is not subject to the event of interest, or who sustains remission that does not require therapy within any reasonable monitoring time window. This is also known as "functional cure".

When the population under study consists of susceptible and non-susceptible (i.e., functional cured) patients, the number of patients who are at risk of the event of interest is smaller. This implies that the time it takes to reach the required number of events with desired level of statistical power is lengthened as the events can only be observed in the subset of susceptible patients.

Another observation that has been made in most randomized immunotherapy trials is the delayed separation of Kaplan-Meier survival curves (Figure 1C, D). Occasionally the separation may occur months after randomization [2]. When the delayed separation of survival curves is present, it violates the fundamental study design assumption of the proportional hazards. The end result is a potential loss of statistical power to demonstrate the difference between two treatment arms.

### Group sequential procedure

Large randomized clinical trials usually take considerable time to enroll patients. In clinical trials involving human subjects, it is imperative to ensure that individuals are not exposed to unsafe or ineffective treatments. Due to the life-threatening nature of cancer and the unmet medical need, it has become common practice to build in group sequential procedures to assess the possibility of terminating the studies early for unexpected large treatment effects or lack of efficacy that suggests a positive result at the end of the study would be extremely unlikely. The former is usually referred to as the interim analysis for superiority, the latter is that for futility.

The most popular approaches were group sequential methods proposed by O’Brien and Fleming [19], Pocock [20], and error-spending functions by Lan and DeMets [21] and Pampallona et al. [22]. Both the Pocock and O’Brien-Fleming procedures require the interim analyses to be performed at more conservative levels, i.e., smaller p-values, to avoid excessive false-positive or false-negative conclusions. The interim analyses are usually specified based on the information fraction, i.e., the proportion of total pre-specified number of events. The error-spending functions are flexible in how many interim analyses are to be conducted and at what times while maintaining the overall experiment-wise error rates. They allow review of unequal increment of the data at unscheduled times.

In an immuno-oncologic randomized clinical trial with long term survival and delayed clinical effect, one needs to reconsider the implementation of conventional interim analyses when the intention is to stop the study early for either positive or futile outcome. If the treatments exhibit delayed clinical benefit, implementation of superiority interim analysis may have smaller stopping probability for a positive outcome whereas futility interim analysis could increase the chance of terminating the study early and erroneously discarding an active agent.

On the other hand, when the treatments under study yield long term survivors, the outcome of interim analyses can not reflect this unique characteristic of the treatments since most of the patients are unlikely to have sufficient follow-ups. Furthermore, the conventional interim analysis at the information fraction of 50% may not be the optimal analysis time since the study duration post interim analysis could take substantially longer due to the decreasing event rate. Therefore, the timing of the interim analysis requires careful plannings such that an interim analysis at a later time may be desired.

### Simulation study

A simulation study was performed in order to evaluate the impact of long term survival and delayed clinical effect introduced by immunotherapy as well as the utility of the interim analysis. A randomized study was first designed using the conventional exponential distribution assumption, i.e., Proportional Hazards Model (PHM), as a benchmark: 512 events were required to detect an overall hazard ratio of 0.75 between two treatment arms using a log-rank test with an experiment-wise two-sided type I error rate of 5% and power of 90%. Furthermore, the accrual duration for 680 randomized patients was assumed to be 34 months. The study was expected to attain 512 events approximately 48 months after the first patient was randomized.

To describe the impact of long term survival and delayed clinical effect, three survival models were introduced. First, a Proportional Hazards Cure Rate Model (PHCRM) added a cured fraction of patients into the proportional hazards model. Under the same assumptions, the proportional cure rates of 10% vs. 18% were incorporated between two treatment arms. The proportional cure rates maintained the risk ratio of the entire population at 0.75 and the power of 90% so the impact on the study duration could be assessed without inducing the loss of power simultaneously. If the magnitudes of the proportional cure rates among non-susceptible patients and the proportional hazards among susceptible patients were different, the power would be impacted either upwards or downwards.

To assess the impact of statistical power under delayed clinical effect, a Non-Proportional Hazards Model (NPHM) was used where the first 3 months were assumed to have hazard ratio of 1 and 0.75 thereafter. This implied the treatment effect of immunotherapy did not take effect until month 3.

The last model, a Non-Proportional Hazards Cure Rate Model (NPHCRM) incorporated both the long term survival and delayed clinical effect by assuming the hazard ratio of 1 during the first 3 months and 0.75 post separation with proportional cure rates of 10% vs. 17%.

Superiority and futility interim analyses with O’Brien-Fleming procedure at the 50% information fraction were also built in for the four models with various combinations of long term survival and delayed clinical effects to evaluate the efficiency of the interim analysis implementation.

Each scenario was evaluated based on 10000 simulations. The simulated power of each design was the proportion of iterations that met the criterion of log-rank test p-value of less than 0.05. The interim stopping probabilities were based on the proportion of iterations among which the estimated efficacy effect crossed the O’Brien-Fleming boundaries. The study duration was calculated based on the median of the simulated durations from 10000 iterations. The median instead of the mean of simulated study durations was chosen in order to prevent potential outliers from skewing the result, i.e., pre-specified number of events not reachable.

## Results

The simulated statistical powers and study durations from PHCRM, NPHM and NPHCRM were compared to those of PHM (Table 1). When a proportion of patients from both arms were long term survivors (10% vs. 18%), PHCRM showed that the study duration was extended from 48 months to 55 months while the power was maintained at 90%. If the anticipated treatment effect of hazard ratio 0.75 was not observed until after the third month, the pre-specified number of 512 events would only provide a 70% power, leading to a 20% absolute loss of power (NPHM). Finally, an immuno-oncologic agent or disease indication that demonstrated both long term survival and delayed clinical effect would lead to an under-powered study, i.e., power of 70%, with extended study duration, i.e., 54 months, as shown in NPHCRM. Note that the shorter study duration and lower long term survival rate under NPHCRM compared to those of PHCRM was due to the first 3 months of delayed clinical effect in the experimental arm under NPHCRM.

**Table 1:** Impact of long term survival and delayed clinical effect on statistical power and study duration

	**PHM**	**PHCRM**	**NPHM**	**NPHCRM**
Cure rate	–	0.10 vs. 0.18	–	0.10 vs. 0.17
Delayed clinical effect (month)	–	–	3	3
Sample size	680	680	680	680
Number of events	512	512	512	512
Hazard ratio (pre- and post- separation)	0.75	0.75	1/0.75	1/0.75
Type I error	0.05	0.05	0.05	0.05
Power	0.90	0.90	**0.70**	**0.70**
Accrual duration (month)	34	34	34	34
Study duration (month)	48	**55**	47	**54**

A study designed to detect a hazard ratio of 0.75 with two-sided type I error rate of 5% and power of 90% requires 512 events under exponential assumption. The study would take 48 months with accrual rate of 20 patients per month for 34 months. When a cured fraction of patients exists in both arms, the study duration is extended to 55 months. A delayed separation in overall survival effect would lead to a loss in power. The presence of both long term survival and delayed clinical effect would lead to an underpowered and lengthy study.

Table 2 illustrated the utility of interim analysis in an immuno-oncologic trial. An interim analysis at the information fraction of 50% was incorporated under the same design assumption. Based on the same accrual rate of 20 patients per month, the total number of randomized patients for the four models ranged from 480 to 540 when 256 events were reached. If the treatment were efficacious, the probability of early termination (PET) in the face of positive outcome under PHM would be 0.25. However, this probability reduced to 0.06 when a 3-month delayed effect was present (NPHM, NPHCRM). Conversely, if a futility interim analysis were implemented in the design, the chance of stopping for futile outcome with an efficacious treatment would be 0.01 under PHM or PHCRM, while NPHM or NPHCRM would lead to an increasing stopping probability of 0.08. With decreasing true positive rate and increasing false negative rate at the interim analysis, the necessity and timing of the interim analysis warrants careful reconsideration.

**Table 2:** Interim stopping probability of superiority or futility with long term survival and delayed clinical effect

	**PHM**	**PHCRM**	**NPHM**	**NPHCRM**
Interim sample size	520	540	480	500
Number of events	256	256	256	256
*PET*_*a*_ (superiority)	0.25	0.25	**0.06**	**0.06**
*PET*_*a*_ (futility)	0.01	0.01	**0.08**	**0.08**

Under the same design assumption, we incorporated an interim analysis at the information fraction of 50%. Based on the same accrual rate of 20 patients per month, the total number of randomized patients for the four models ranged from 480 to 540 when 256 events were reached. When a superiority interim analysis using O’Brien-Fleming boundaries was built into the study design, the probability of early termination when the agent was active (*PET*_*a*_), i.e., true positive rate, at the interim analysis under exponential decay was 0.25. The *PET*_*a*_ reduced to 0.06 when the delayed clinical effect of 3 months was present. If a futility interim analysis was incorporated, the *PET*_*a*_ (false negative rate) increased from 0.01 to 0.08.

In addition to the challenges that immuno-modulating agents created with regards to the study design and interim monitoring, two additional issues were being considered. The first was the choice of response assessment criteria that accounted for long term benefits of these agents, and the second was the safety analysis algorithm that efficiently identified inflammatory adverse events.

### Measurement of anti-tumor response: Immune-related response criteria (irRC)

Anticancer activity derived from chemotherapeutic agents traditionally has been assessed using modified World Health Organization (WHO) [23] or Response Evaluation Criteria in Solid Tumours (RECIST) criteria [24,25]. These guidelines standardized the efficacy assessment and facilitated comparison between clinical studies. However, the underlying operating assumption of these guidelines lies in the effect of chemotherapeutic agents on suppressing early tumor growth. Therefore, an appearance of a new lesion signals progressive disease (PD). The treatment is recommended to be terminated once PD is detected.

While these guidelines have served us well in evaluating chemotherapeutic agents, recent research experience in immuno-oncology has indicated that the clinical benefit from immunotherapy might extend beyond that of cytotoxic agents. For example, stable disease or responses to immunotherapy may occur after conventional PD due to clinically insignificant new lesions in the presence of other responsive lesions and reduction of the total tumor burden. Therefore, discontinuation of immunotherapy at the first sight of PD may not be appropriate in some cases. In addition, measurable antitumor activity may take longer for immunotherapies than for cytotoxic agents, and durable stable disease (SD) may represent meaningful antitumor activity. A novel set of anti-tumor assessment criteria, irRC, was therefore proposed based on WHO and RECIST criteria and has been applied to a series of phase II and phase III ipilimumab clinical trials [2,3,5,26-28]. The essence of the irRC is the incorporation of the concept of total measurable tumor burden in the assessment of the anti-tumor activity. Readers can refer to Wolchok et al. [29] for the detailed description of the irRC guidelines. When the irRC is implemented, each patient could potentially have two progression dates: an earlier date determined by the conventional criteria and a later one by the irRC. While the time to event analysis can be applied to the new irRC by considering the irPD (immune-related progressive disease) as an event, the depth of the tumor burden reduction over time can also be studied via longitudinal data analyses.

One of the biggest challenges of using irRC in assessing immunotherapeutic responses is the uniformity of implementation among treating physicians. For example, when treatments are available for patients with PD due to the appearance of new lesions per conventional modified WHO or RECIST criteria, treating physicians may incline to switch the patients to other available therapies prior to observing delayed benefit rather than prolonging the time on the current experimental therapies. A large number of physicians and sites are usually involved in randomized clinical trials. This will render the interpretation of response assessment results difficult since different criteria were implemented in determining the clinical benefit within the same study.

Despite the implementation challenge outlined above, the potential need of a new set of guidelines for immuno-oncologic agents can not be overlooked. The utility of irRC will be studied in depth in the near future with richer data collection when the criteria are more widely adopted.

### Measurement of adverse events

#### Immune-related adverse events (irAE)

Consistent with the idea that immunotherapies fight cancer by augmenting the immune response, most adverse events are expected to be inflammatory in nature. As such, immunotherapy associated adverse events differed in character from those observed in melanoma patients treated with other therapies.

To better identify adverse events likely due to immuno-oncologic agents such as ipilimumab, a method was developed to quantify immune-related adverse events (irAEs) [30]. IrAEs were defined as adverse events of unknown etiology, consistent with an immune phenomenon, considered by the investigator to be related to treatments. A list of pre-specified set of Medical Dictionary for Regulatory Activities (MedDRA) preferred terms of likely immune system origin was chosen a priori. These terms belong to six particular system organ classes: Enterocolitis, Hepatotoxicity, Dermatitis, Neuropathy, Endocrinopathy, and Other. The adverse events prospectively identified by the investigators based on a blinded assessment of individual events were categorized based on these organ systems in the irAE analysis.

In contrast to regular safety analyses, the irAEs were able to better discriminate between adverse events caused by ipilimumab versus those due to other factors. Nevertheless, adverse events being classified as irAEs continued to be observed among patients treated with other therapies. The incidence of irAE among these patients should be closed to zero if the irAE methodology truly captured only events with immune system origin. A greater specificity was needed to accurately identify immunotherapy-specific events and accurately characterize the safety profile of immuno-oncologic agents such as ipilimumab.

#### Immune-mediated adverse reactions (imAR)

A new method was developed for the identification of immune-mediated adverse reactions in order to determine which adverse events were truly immune-related [31]. This method was used to retrospectively evaluate adverse events reported in ipilimumab studies [2,3]. This methodology differed from irAE method in that: (1) similar adverse event records were grouped into contiguous events; (2) grade 1 events were excluded unless the events are part of the contiguous events of higher grades, (3) hepatic laboratory abnormalities, i.e., grade 2+ AST/ALT elevations, were included in the assessment of hepatic imAR, and (4) the assessment was performed retrospectively by taking into account the information such as the use of concomitant medications, and duration of the adverse events.

Similar to irAE, the description of the adverse event had to first include at least one of a set of pre-specified preferred terms in order for an adverse event to be considered an imAR. The adverse event records were then reviewed against a predefined list of criteria for attribution as an imAR specific to each of the same system organ classes defined in the previous section (i.e., Enterocolitis, Hepatotoxicity, Dermatitis, Neuropathy, Endocrinopathy, and Other). The determination of imAR was based on the rule of elimination. If any of the exclusion criteria was met, e.g., administration of intervening treatment with chemotherapy proximal to the event known to cause irAE, or rapid resolution of the adverse event (< 1 week) without initiating concomitant immunosuppressive agents, the event was adjudicated as not attributed to ipilimumab. Hence, these adverse events would not be characterized as an imAR.

For the identification of imARs in ipilimumab [2,3], each adverse event was adjudicated in a blinded fashion using the above criteria by two independent physicians. Any remaining disagreements would trigger a review by a third physician; the final adjudication was based on a majority vote. The findings from the analyses indicated that the imAR method yielded great sensitivity and specificity as the incidence rate of imAR in non-ipilimumab arms fell close to zero [30,31].

## Discussion

Statistical models for time to event analysis typically assume that everybody in the study population is susceptible to the event of interest and will eventually become an event. In recent years, due to the innovative medical advancement, new treatments such as immunotherapies have yielded long term survivors. Some people in the population may be considered cured or non-susceptible. Failing to account for these subjects in the study design could potentially impact the process of drug development and resource planning. Furthermore, the newly introduced immuno-oncologic agents may not show early benefit due to their mechanism of action. This leads to a delayed separation in Kaplan-Meier curves in time to event analysis.

In this article, the consequence on statistical power and study duration in the presence of long term survival and delayed clinical effects when the study was designed based on the conventional assumption of exponential distribution was thoroughly discussed. For illustration purpose, the clinical trial example provided in this article assumed a 3-month delayed separation in overall survival curves. If the separation occurred at a later time, the loss of power would become more severe. Note that the statistical power was also dependent on the magnitude of the treatment effect post the survival curve separation. If the true treatment effect post separation exceeds expectations, the statistical power will improve based on the magnitude of the treatment effect.

In the simulation study, the proportional cure rates were assumed in order to evaluate the study duration without introducing power change. If a PHCM study was designed and in reality the cure only existed in the experimental arm, this design would yield an over-powered study. On the other hand, if the cure existed in the control arm only, or the magnitude of the proportional cures was weaker than that of proportional hazards, the study will be under-powered. Another element that dictates the study duration is the magnitude of the cure rate. The bigger the subset of non-susceptible population in a study, the longer it would take to reach the pre-specified number of events.

Furthermore, the objective of group sequential procedure is to terminate the study in the face of either positive or futile result prior to the completion of the study. When the treatment under study possessed the unique immuno-oncologic characteristics, the necessity of the interim analyses, for superiority or futility, need to be considered with caution. If the interim analyses are to be built in, it is imperative to implement them at the optimal time points, accounting for clinical, statistical and operational considerations.

One area that was not touched upon in this article was the time to event analysis with long term survival and delayed clinical effect. The most commonly used statistical methods for time to event analyses have been the log-rank test and Cox regression analysis. These standard analyses have maximal statistical power under the proportional hazards assumption. However, they do not emphasize the treatment effect on the long-term survivors, which may be of primary interest. In some occasions, researchers may be interested in estimating the cured fraction. One way of demonstrating long term survival is to present the survival rates at given time points, such as one-year or two-year OS rates. If such analysis is to be conducted, one should ensure the minimum follow-up duration is at least as long as the time point of interest to reduce the number of censored observations. For example, a minimum follow-up of two years will yield robust estimates of OS rates up to two years.

Cure rate models have been a popular topic within statistical literature, these models can be a useful tool to analyze and describe time to event data [32-35]. Nevertheless, the use of such models should be restricted to problems in which strong biological evidences suggest the presence of a cured fraction. One important aspect that needs to consider when speaking of ’cure’ is whether the study provides sufficient follow-up. The leveling off of the Kaplan–Meier curve to nonzero proportions, the presence of a long and stable plateau with a heavy censoring at the tail may provide evidences of the presence of patients with functional cure. A robust estimate of cure fraction can only be determined with sufficiently long follow-ups [3]. Therefore, one should consider enrolling a smaller number of patients in an event-driven study to ensure sufficient follow-ups on all patients.

Statistical methodology such as weighted log-rank test (WLR) [8,36,37] also exists to account for the delayed separation of Kaplan-Meier curves. When the delayed clinical effect is present, the WLR test is much more powerful than the conventional non-parametric log-rank test. Thus, it is an appropriate alternative to be considered as an exploratory analysis.

The newly proposed efficacy (irRC) and safety (irAE and imAR) analyses were also discussed when the treatments under study were immunotherapies. The irRC modified the widely used WHO or RECIST criteria by considering the total measurable tumor burden in the evaluation of anti-tumor activities. The new criteria allow continuation of immunotherapy treatment in the face of clinically insignificant new lesions in the presence of responsive lesions, leading to potential late responses. Ultimately, a proportion of patients who would have fulfilled conventional criteria for progressive disease was shown to have long term survival rate similar to that from the conventionally defined responding patients. The challenge of implementing irRC, similar to any assessment guidelines, is to ensure the data interpretability by applying uniform evaluation across sites and investigators in clinical trials. The utility of irRC will be studied in depth in the near future with richer data collection when the criteria are more widely adopted.

Two new safety analysis methods were also proposed to account for the characteristics of immuno-oncologic therapies. While the prospective irAE method facilitated prompt and effective management of adverse events, the retrospective imAR method captured truly immune-related events. If these new safety analysis methods are to be implemented, the criteria of irAE/imAR identification require careful considerations based on biological and clinical evidence for the immunotherapies under study.

## Conclusions

When designing randomized clinical studies with immunotherapies, the simulation study indicated that the conventional study design with exponential assumption could lead to an underestimation of either statistical power or study duration in the presence of delayed clinical effect or long term survival. The necessity and timing of superiority or futility interim analysis also required careful consideration due to decreasing true positive rate or increasing false negative rate. New efficacy and safety analyses were proposed to account for the mechanism of action in immunotherapies. These findings have important implications for clinical trial researchers aiming to optimize the drug development process and bring the novel treatments to cancer patients in need.

## Competing interests

The author is an employee of Bristol-Myers Squibb.

## Author’s information

The author is director in oncology biostatistics and the lead biostatistician of Yervoy (Ipilimumab) at Bristol-Myers Squibb. He is also an Adjunct Assistant Professor of Biostatistics at Columbia University, New York, NY, USA.
